# Prognostic impact of hand-foot skin reaction in regorafenib-treated adult-type diffuse gliomas: A multicenter Turkish Oncology Group study

**DOI:** 10.1038/s41598-025-19899-3

**Published:** 2025-10-16

**Authors:** Safa Can Efil, Burak Bilgin, Irem Bilgetekin, Kaan Helvaci, Teoman Sakalar, Erdinc Nayir, Fatma Esra Erdem Palaz, Ali Kaan Guren, Sedat Biter, Taha Koray Sahin, Ertugrul Bayram, Neyran Kertmen, Ahmet Melih Arslan, Esra Asık, Orhun Akdogan, Ozkan Alan, Evren Fidan, Ozan Yazici, Elif Sahin, Engin Kut, Ceren Mordag Cicek, Sema Turker, Nagihan Kolkiran, Gamze Gokoz Dogu, Mustafa Sahbazlar, Aziz Batu, Bilgesah Kilictas, Mesut Yilmaz, Nejat Emre Oksuz, Mehmet Artac, Sinem Akbas, Efnan Algin, Yuksel Urun, Mehmet Ali Nahit Sendur, Bulent Yalcin

**Affiliations:** 1 Department of Medical Oncology, Ankara Bilkent City Hospital, Ankara, Turkey; 2 Department of Medical Oncology, Memorial Ankara Hospital, Ankara, Turkey; 3 Department of Medical Oncology, Kahramanmaras Sutcu Imam University, Kahramanmaras, Turkey; 4 Department of Medical Oncology, Toros University, Mersin, Turkey; 5 Department of Medical Oncology, Mersin University, Mersin, Turkey; 6 Department of Medical Oncology, Marmara University, Istanbul, Turkey; 7 Department of Medical Oncology, Cukurova University, Adana, Turkey; 8 Department of Medical Oncology, Hacettepe University, Ankara, Turkey; 9 Department of Medical Oncology, Dokuz Eylul University, Izmir, Turkey; 10 Department of Medical Oncology, Karadeniz Technical University, Trabzon, Turkey; 11 Department of Medical Oncology, Gazi University, Ankara, Turkey; 12 Department of Medical Oncology, Cerrahpasa Istanbul University, Istanbul, Turkey; 13 Department of Medical Oncology, Kocaeli City Hospital, Kocaeli, Turkey; 14 Department of Medical Oncology, Manisa City Hospital, Manisa, Turkey; 15 Department of Medical Oncology, Pamukkale University, Denizli, Turkey; 16 Department of Medical Oncology, Bahcesehir University, Istanbul, Turkey; 17 Department of Medical Oncology, Manisa Celal Bayar University, Manisa, Turkey; 18 Department of Medical Oncology, Umraniye Training and Research Hospital, Istanbul, Turkey; 19 Department of Medical Oncology, Necmettin Erbakan University, Konya, Turkey; 20 Department of Medical Oncology, Sakarya Training and Research Hospital, Sakarya, Turkey; 21 Department of Medical Oncology, Ankara University, Ankara, Turkey; 22 Department of Medical Oncology, Koc University, Istanbul, Turkey

**Keywords:** Adult-type diffuse gliomas, Glioblastoma, Regorafenib, Hand–foot skin reaction, Cancer, Oncology

## Abstract

Glioblastoma, IDH-wildtype is the most aggressive primary brain tumor in adults, and treatment options for recurrent disease are limited. Regorafenib, an oral multikinase inhibitor, has shown efficacy in glioblastoma at first recurrence, but its role in later recurrences and in other adult-type diffuse gliomas remains unclear. This multicenter retrospective study aimed to evaluate the safety and efficacy of regorafenib and to identify prognostic factors in patients with adult-type diffuse gliomas, including glioblastoma, who were treated at the second or subsequent recurrences. A total of 68 patients from 22 institutions were analyzed. The median overall survival (OS) was 3.84 months, and the median progression-free survival was 2.46 months. The objective response rate was 13%, and the disease control rate was 48%. Adverse events occurred in 80.9% of patients, most commonly fatigue, anemia, and elevated transaminases. Hand–foot skin reaction (HFSR) of any grade was observed in 36.4% of patients and was associated with significantly improved OS (HR: 0.41; p = 0.005). Regorafenib demonstrated a manageable safety profile and modest activity in this heavily pretreated population. The development of HFSR emerged as a potential prognostic marker for treatment benefit. Taken together, our findings support further exploration of regorafenib in this setting and suggest that HFSR may serve as a practical marker to guide treatment continuation.

## Introductıon

Glioblastoma multiforme (GBM), recently reclassified as glioblastoma IDH-wild type, is the most common and aggressive central nervous system (CNS) tumor in adults^1^. The standard approach, known as the Stupp protocol, combines maximal safe tumor resection with radiotherapy and temozolomide; however, recurrence remains almost inevitable^2^. Currently, there is no standardized treatment strategy for recurrent adult-type diffuse gliomas, including glioblastoma. If tumor location and patient characteristics permit, re-resection or re-irradiation may be considered; however, these options are feasible in only a limited number of patients, and most ultimately receive systemic therapy^3,4^. Patients with methylation of the O6-methylguanine-DNA methyltransferase (MGMT) promoter may benefit from rechallenge with high-dose temozolomide^5^, and nitrosoureas such as lomustine are commonly used despite their modest efficacy^6^. These tumors are characterized by the expression of vascular endothelial growth factor (VEGF) receptors (VEGFRs) and exhibit marked angiogenesis^7,8^, providing a rationale for anti-angiogenic agents such as bevacizumab^9^. Moreover, immune checkpoint inhibitors including pembrolizumab and nivolumab have been evaluated in patients with recurrent glial tumors; however, none has demonstrated a significant clinical benefit.^10,11^. The poor outcomes observed in patients with recurrent adult-type diffuse gliomas underscore the urgent need for novel systemic therapies.

Regorafenib is an oral multikinase inhibitor that inhibits pathways associated with oncogenesis (C-KIT, RET, BRAF), neovascularization (VEGFR 1–3), and the tumor microenvironment (platelet-derived growth factor receptor [PDGFR], fibroblast growth factor receptor [FGFR])^12^. The therapeutic effectiveness of regorafenib has been demonstrated for metastatic colorectal cancer (CRC)^13^, advanced gastrointestinal stromal tumour (GIST)^14^, and advanced hepatocellular carcinoma (HCC)^15^. It has also been linked to the induction of lethal autophagy, potentially limiting tumor progression in glioblastoma cell lines^16^. The efficacy of regorafenib in glioblastoma patients with disease recurrence after first-line treatment was evaluated in the phase II REGOMA trial^17^. In this study, regorafenib was compared with lomustine and demonstrated a median overall survival (OS) benefit (7.4 vs. 5.6 months) and a higher disease control rate (DCR) (44% vs. 20%), favoring regorafenib. However, adverse events were reported more frequently in the regorafenib arm, but did not adversely affect health-related quality of life^18^. Following these positive results, regorafenib was included in international guidelines as a preferred treatment option for patients with recurrent glioblastoma^19^.

Following the REGOMA trial, subsequent observational and retrospective studies^20–22^ assessed the efficacy and safety of regorafenib after first recurrence of glioblastoma; however, its use in second or subsequent recurrences remains insufficiently evaluated. Moreover, data on the use of regorafenib in other recurrent adult-type diffuse gliomas beyond glioblastoma are limited. Therefore, this multicenter real-world study aims to assess the efficacy and safety of regorafenib in patients with adult-type diffuse gliomas, including glioblastoma at second or subsequent recurrences, and to identify clinicopathological factors associated with survival. In particular, we were interested in exploring the prognostic significance of hand–foot skin reaction (HFSR), a common adverse effect of regorafenib, as a potential clinical marker of treatment response.

## Methods

### Patients population

This retrospective analysis included patients with adult-type diffuse gliomas who received regorafenib at second or later recurrences, based on data collected from 22 institutions in Turkey between April 2020 and October 2024. Demographic, clinicopathological, laboratory, and radiologic data were extracted from institutional medical database. Inclusion criteria were: histologically confirmed adult-type diffuse gliomas, radiologically confirmed second or subsequent tumor recurrence as defined by the Response Assessment in Neuro-Oncology (RANO) Working Group^23^; age ≥ 18 years; and an Eastern Cooperative Oncology Group performance status (ECOG-PS) ≤ 3. Adult-type diffuse gliomas included glioblastoma, IDH-wildtype; astrocytoma, IDH-mutant; and oligodendroglioma, IDH-mutant. Although glioblastoma is currently defined exclusively as IDH-wildtype according to the 2021 WHO Classification of CNS Tumors^1^, we also included IDH-mutant glioblastomas in our cohort. This decision was necessitated by the retrospective and multicenter design of the study, and 7 patients had been classified as glioblastoma, IDH-mutant according to the former WHO terminology at the time of diagnosis. Patients with incomplete follow-up data or contraindications to regorafenib were excluded.

IDH mutation status was assessed by immunohistochemistry (IHC) using anti-IDH1 antibody. MGMT promoter methylation was evaluated by polymerase chain reaction (PCR)-based methylation-specific assay. Molecular testing was performed on available tumor tissue samples; however, MGMT promoter methylation analysis was feasible only in a subset of patients (n = 17).

### Regorafenib treatment and follow-up

Regorafenib was administered orally for 21 days of a 28-day cycle at initial doses of 80–160 mg, determined at the investigator’s preference, with a target final dose of 160 mg. Since the primary aim of this retrospective study was to investigate prognostic factors associated with survival, detailed dose information was not initially collected, and therefore cumulative dose analyses could not be performed. Dose reductions, delays, or interruptions were made based on patient tolerance and adverse events, which were evaluated according to the National Cancer Institute’s Common Terminology Criteria for Adverse Events (CTCAE), version 5.0. HFSR was routinely assessed and graded by treating medical oncologists during scheduled visits. Routine dermatological examinations were not mandated; however, dermatology consultations were sought in cases of diagnostic uncertainty or when additional management was required. Clinical and laboratory follow-up was conducted at 2- to 4-week intervals, and radiological follow-up with gadolinium-enhanced brain MRI was performed at 8- to 12-week intervals. Radiologic tumor response was assessed using the RANO Working Group criteria^23^.

### Endpoints and statistical analysis

The main endpoint was OS while the additional endpoints included progression-free survival (PFS), objective response rate (ORR), DCR and safety data. OS was defined as the duration from the initiation of regorafenib to the time of death. PFS was defined as the time from the initiation of regorafenib to radiological progression or death. Radiological progression was evaluated according to the RANO criteria using gadolinium-enhanced brain MRI, performed at 8- to 12-week intervals and assessed by experienced neuroradiologists. In cases where pseudoprogression or radiation necrosis was suspected, shorter-interval MRI follow-up was undertaken to facilitate distinction from true progression. No patient underwent biopsy or re-operation for histopathological confirmation of progression. ORR was defined as partial response (PR) and complete response (CR) based on RANO criteria. DCR was defined as the combination of PR, CR, and stable disease (SD). Statistical analyses were performed using SPSS version 26.0 and R version 4.5.0. Patient characteristics were presented as medians with ranges for continuous variables and as frequencies and percentages for categorical variables. Chi-square test was used to compare categorical variables. The log-rank test was used to investigate differences in survival outcomes, and Kaplan–Meier survival estimates were calculated. Cox regression analyses were performed to identify variables associated with OS and PFS. A two-sided p-value of < 0.05 was considered statistically significant.

### Statistical power analysis

Given the retrospective design, no a priori sample size calculation was performed. To quantify detectability and achieved sensitivity post hoc, we used the Schoenfeld approximation for Cox proportional hazards models (two-sided α = 0.05). The calculations were based on the total cohort size (n = 68), the proportion of patients across binary predictors (e.g., ECOG status, HFSR occurrence), and the number of observed survival events (53 for OS and 57 for PFS). For each covariate assessed in the multivariable Cox regression, we estimated the minimum detectable hazard ratio (MDHR) at 80% power as well as the achieved power for the observed effect sizes.

### Ethics approval

The study was conducted in accordance with the Declaration of Helsinki, and the protocol was approved by the Scientific and Ethical Evaluation Board for Medical Research at Ankara Bilkent City Hospital (No:1–25-1258). In accordance with the retrospective and observational design of this study, the requirement for informed consent was formally waived. Detailed information on the relevant ethical guidelines is available on the Institutional Review Board’s official website (https://ankarasehir.saglik.gov.tr/TR-494394/tabed-yonerge.html).

## Results

### Baseline characteristics of patients and data on regorafenib treatment

This study included a total of 68 patients diagnosed with adult-type diffuse gliomas. Baseline patient characteristics and data on regorafenib treatment are presented in Table 1. The median age at initiation of regorafenib treatment was 57 years (range: 27–74) with 58.8% of patients being male. The majority of the patients (57.4%) exhibited an ECOG-PS of 2–3. IDH mutations were detected in 13 patients, and MGMT promoter methylation was confirmed in 4 of 17 assessed patients. Among the patients, 55 (80.9%) were diagnosed with glioblastoma, IDH-wildtype, 7 (10.3%) had glioblastoma, IDH-mutant, 4 (5.9%) were identified with astrocytoma, IDH-mutant, grade 3, and 2 (2.9%) were diagnosed with oligodendroglioma, IDH-mutant, grade 3. Complete tumor resection was performed in 54.7% of patients at initial surgery. First-line treatment in most patients (94.1%) consisted of postoperative temozolomide with concurrent radiochemotherapy followed by adjuvant temozolomide.

**Table 1:** Patient baseline characteristics and treatment details with regorafenib.

Characteristics	n (%)
All patients	68 (100)
Age at initiation of regorafenib, median (min–max)	57 (27–74)
Age	
< 65 years	54 (79.4)
≥ 65 years	14 (20.6)
Gender	
Female	28 (41.2)
Male	40 (58.8)
ECOG-PS at initiation of regorafenib	
0–1	29 (42.6)
2–3	39 (57.4)
Histology	
Glioblastoma, IDH-wild type	55 (80.9)
Astrocytoma, IDH-mutant, grade 3	4 (5.9)
Oligodendroglioma, IDH-mutant, grade 3	2 (2.9)
Glioblastoma, IDH-mutant^#^	7 (10.3)
IDH status	
Mutated	13 (19.1)
Wild	55 (80.9)
Type of initial surgery	
Complete resection	39 (57.4)
Partial resection or biopsy only	29 (42.6)
First-line treatment	
CRT (with TMZ) follewed by adjuvant TMZ	64 (94.1)
CRT only	3 (4.4)
RT follewed by adjuvant TMZ	1 (1.5)
Line of regorafenib therapy	
3	58 (85.3)
4	8 (11.8)
5	2 (2.9)
Surgery before regorafenib	
No	67 (98.5)
Yes	1 (1.5)
Radiation before regorafenib	
No	54 (79.4)
Yes	14 (20.6)
Concomitant corticosteroid	
No	25 (36.8)
Yes	43 (63.2)
Initial dose of regorafenib	
80 mg	40 (58.8)
120 mg	19 (27.9)
160 mg	9 (13.2)
No. of regorafenib cycle, median (min–max)	2 (1–7)
Dose reduction due to adverse events	22 (32.4)
Best response based on RANO criteria*	
CR	0 (0.0)
PR	6 (13.0)
SD	16 (35.0)
PD	24 (52.0)
Reason for discontinuation of regorafenib†	
Progression or death	53 (93.0)
Advers events	4 (7.0)
No. of treatment lines after regorafenib	
Regorafenib is ongoing	11 (16.2)
Regorafenib is the last line	50 (73.5)
Received 1 line of treatment after regorafenib	6 (8.8)
Received 2 line of treatment after regorafenib	1 (1.5)

CR: Complete response, CRT: Chemoradiotherapy, ECOG-PS: Eastern Cooperative Oncology Group performance score, PD: Progressive disease, PR: Partial response, RT: Radiotherapy, SD: Stable disease, TMZ: Temozolomid.

^#^ Included as glioblastoma, IDH-mutant according to previous WHO classifications.

* Not evaluable because death occurred in 20 patients, including 2 patients due to an adverse event.

^†^Regorafenib is ongoing in 11 patients.

Regorafenib was administered as third-line therapy after the second recurrence in most patients (85.3%), while 8 patients received it as fourth-line, and 2 patients as fifth-line treatment. The median number of regorafenib treatment cycles was 2 (range: 1–7). Concomitant corticosteroid use was observed in 63.2% of patients at the initiation of regorafenib treatment. Seven patients initiated subsequent therapy due to disease progression following regorafenib.

### Survival analyses and treatment response

**Fig. 1:**
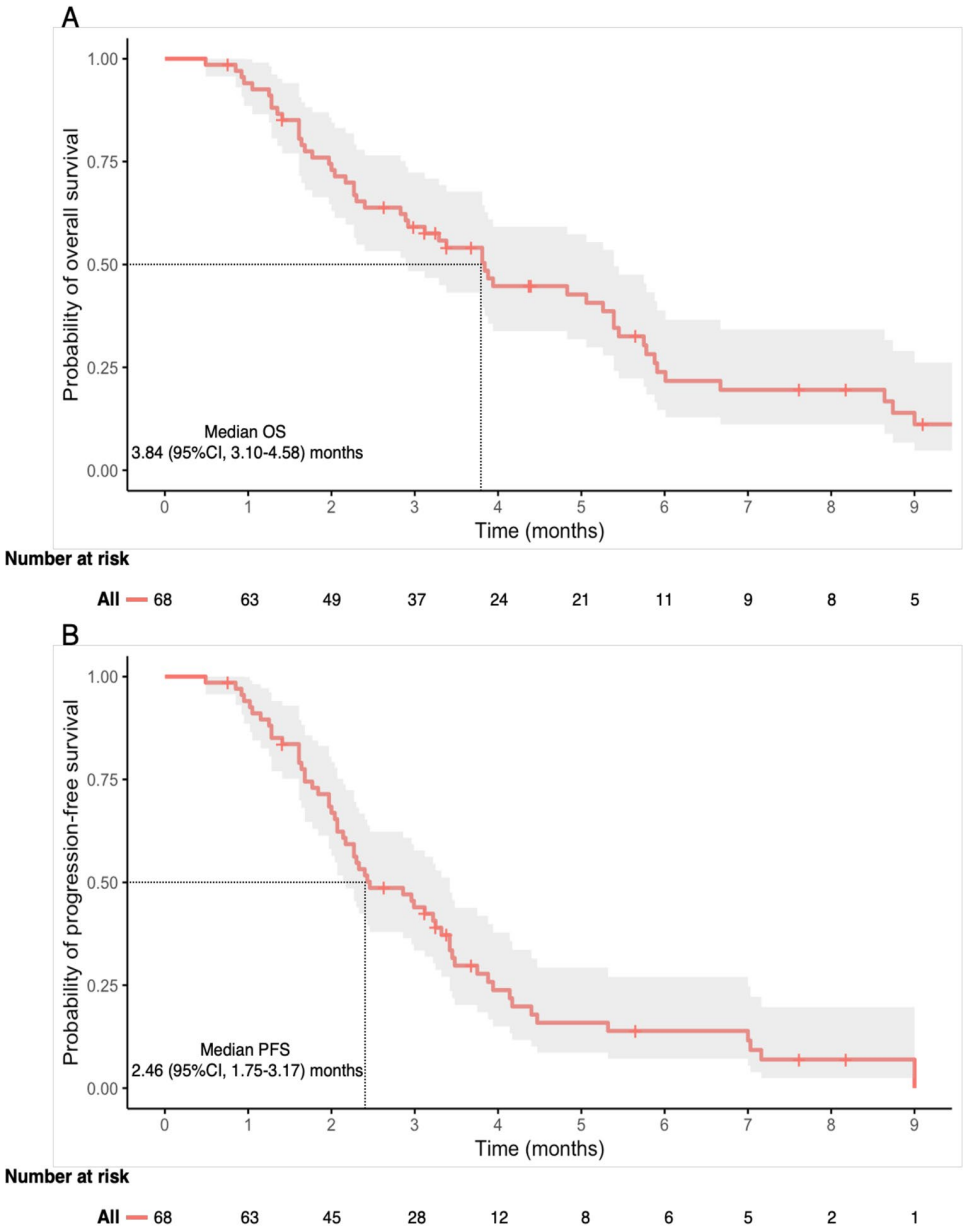
Kaplan–Meier survival curves for the entire study cohort. (**A**) Overall survival. (**B**) Progression-free survival. Shaded areas represent 95% confidence intervals.

After a median follow-up of 9.10 months (95% CI, 7.11–11.09), 57 patients had progressed or died, while 15 remained alive, including 11 who were still receiving regorafenib. The median OS for all patients was 3.84 months (95% CI, 3.10–4.58), and the median PFS was 2.46 months (95% CI, 1.75–3.17) (Fig. 1). According to the RANO criteria, the response to regorafenib treatment was evaluable in 46 patients. Of these patients, none achieved a CR; 13% achieved a PR, 35% had SD, and 52% had PD. The ORR (CR + PR) was 13%, and the DCR (CR + PR + SD) was 48%.

**Fig. 2:**
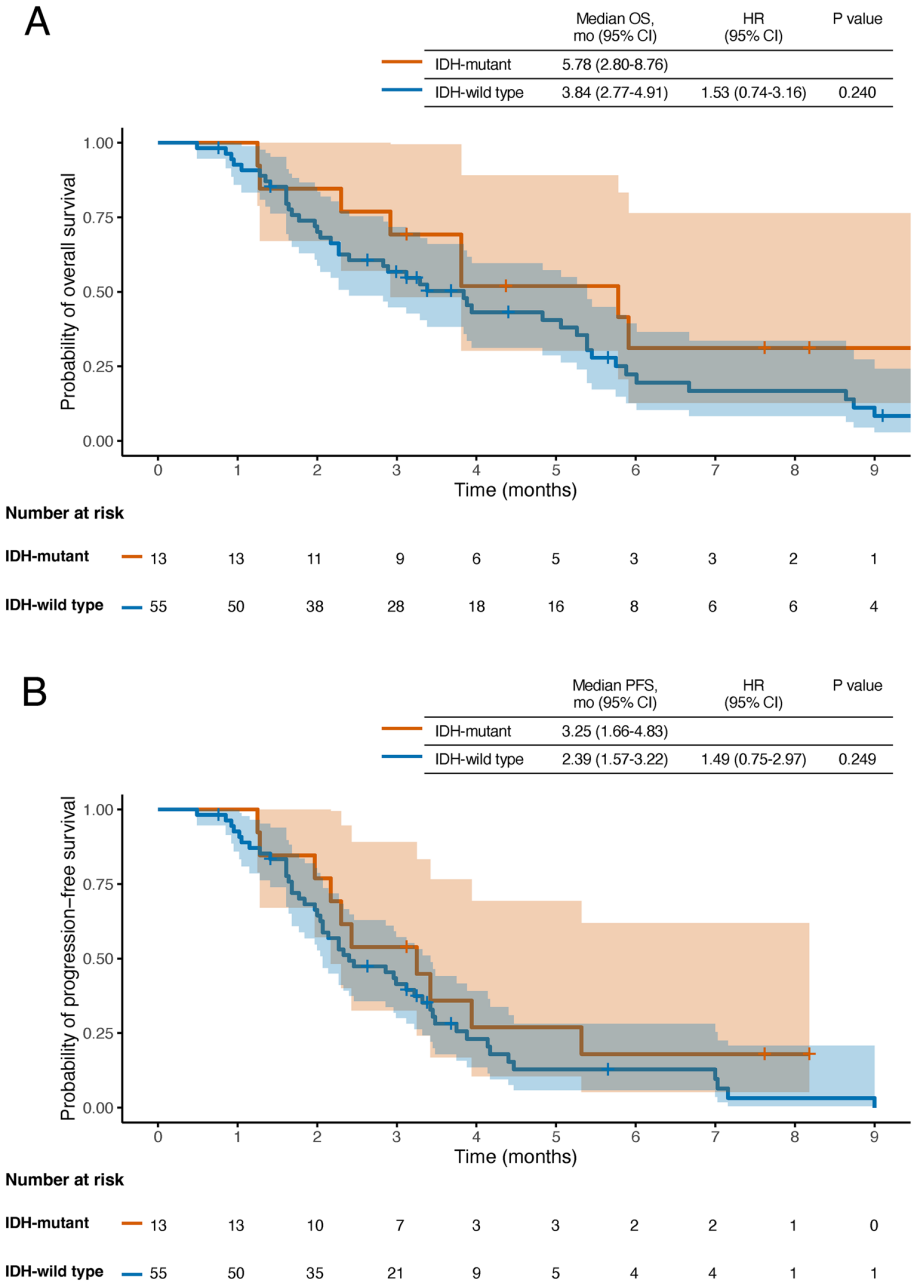
Kaplan–Meier survival curves for overall survival according to the IDH status. (**A**) Overall survival. (**B**) Progression-free survival. Shaded areas represent 95% confidence intervals. Survival distributions were compared using the log-rank test.

Given the prognostic relevance of IDH status, we performed additional subgroup analyses stratified by IDH status. Patients with IDH-wildtype glioblastoma had poorer median OS and PFS compared with those with IDH-mutant gliomas; however, these differences did not reach statistical significance (median OS: 3.84 vs. 5.78 months, p = 0.240; median PFS: 2.39 vs. 3.25 months, p = 0.249) (Fig. 2).

Furthermore, to explore the potential impact of treatment-related factors, we analyzed initial dosing and dose modification patterns. Patients who started regorafenib at 80 mg (58.8% of patients) had a shorter median OS compared with those who started at 120–160 mg (41.2% of patients) (3.38 vs. 5.45 months, p = 0.225), while patients who required dose reductions due to adverse events showed a trend toward longer OS compared with those without dose reduction (5.45 vs. 2.92 months, p = 0.060), although not statistically significant.

### Advers events and safety data

Adverse events (AEs) of any grade occurred in 55 patients (80.9%), while grade 3–4 AEs were documented in 15 patients (22.1%). The most commonly reported AEs of any grade were fatigue (72.7%), anemia (43.9%), and elevated transaminases (42.4%). The most common grade 3–4 AEs included fatigue (9.1%), neutropenia (4.5%), and thrombocytopenia (4.5%) (Table 2). HFSR of any grade was observed in 24 patients (36.4%) and most of which were grade 1–2 (23 patients, 34.9%). Patients with or without HFSR had comparable baseline clinicopathological characteristics; although 54.2% of patients with HFSR had ECOG-PS 0–1 compared to 38.1% without HFSR, this difference was not statistically significant (p = 0.206) (Supplementary Table S1). During the follow-up period, regorafenib dose reduction due to adverse events was required in 22 patients (32.4%). Interruption or delay of a regorafenib cycle was observed in 16 patients (23.5%). Discontinuation of regorafenib treatment due to AEs was observed in 4 patients (5.9%). There were no regorafenib-related deaths.

**Table 2:** Adverse events during regorafenib treatment.

	Any Grade n,(%)	Grade 1–2 n,(%)	Grade 3–4 n,(%)
Anemia	29 (43.9)	29 (43.9)	0 (0)
Neutropenia	25 (37.8)	22 (33.3)	3 (4.5)
Lymphopenia	23 (34.9)	22 (33.4)	1 (1.5)
Thrombocytopenia	24 (36.4)	21 (31.9)	3 (4.5)
Transaminases increased	28 (42.4)	26 (39.4)	2 (3)
Bilirubin Increase	13 (19.7)	13 (19.7)	(0)
Hypertension	11 (16.9)	9 (13.8)	2 (3.1)
Fatigue	48 (72.7)	42 (63.6)	6 (9.1)
Diarrhea	20 (30.2)	18 (27.2)	2 (3)
Hand-foot skin reactions	24(36.4)	23 (34.9)	1 (1.5)

As expected, patients who developed HFSR required dose reductions more frequently compared with those without HFSR (54.2% vs. 21.4%, p = 0.007). In addition, patients with HFSR tended to receive a longer duration of regorafenib therapy, reflected by a higher median number of treatment cycles (3 vs. 2 cycles, p = 0.054) (Supplementary Table S1).

### Prognostic factors associated with PFS and OS

**Fig. 3:**
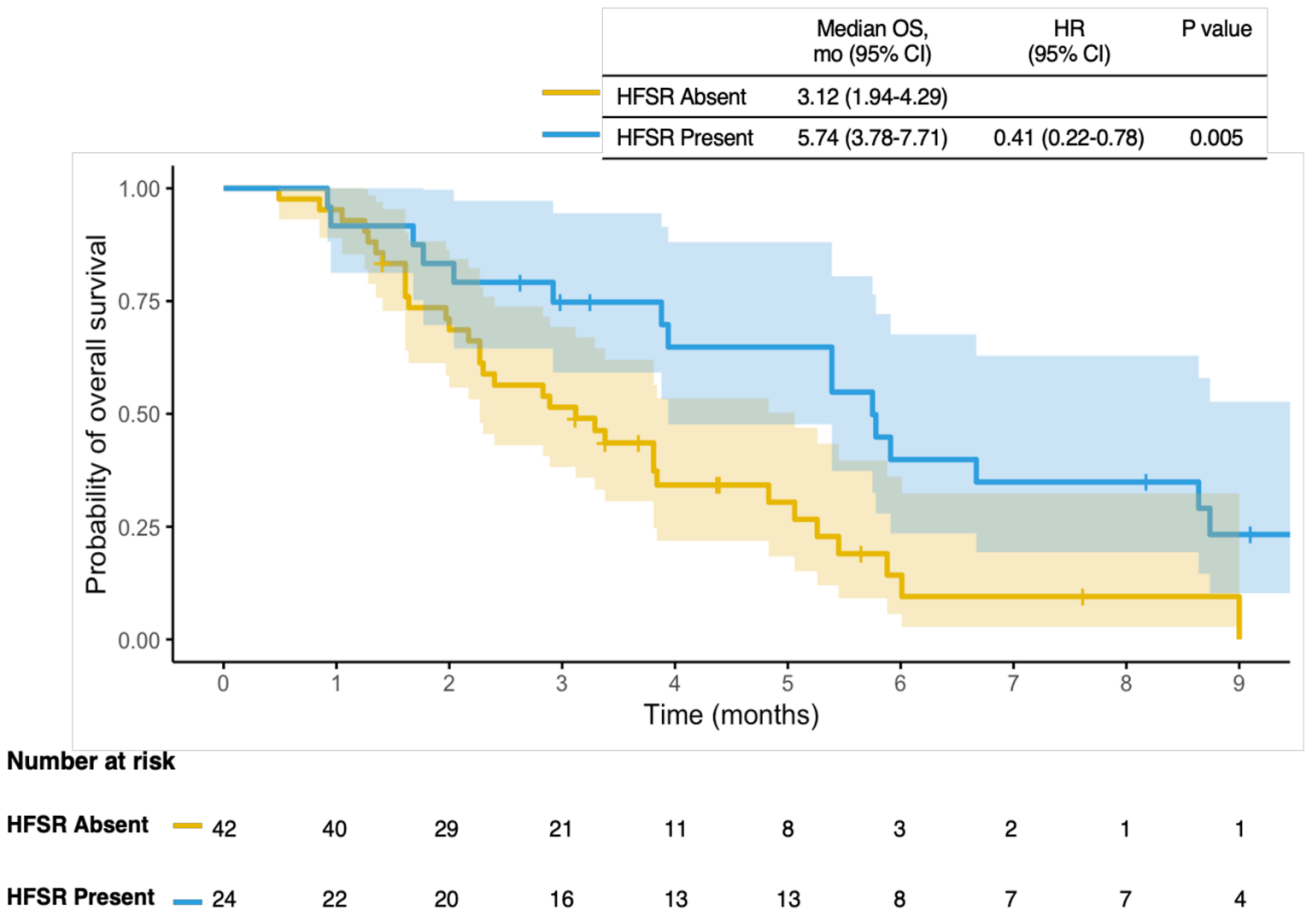
Kaplan–Meier survival curves for overall survival according to the development of hand–foot skin reaction (HFSR). Shaded areas represent 95% confidence intervals. Survival distributions were compared using the log-rank test.

We performed Cox regression analysis to investigate clinicopathologic factors (age, gender, ECOG-PS, histology, IDH status, type of initial surgery, line of regorafenib treatment, concomitant corticosteroid use, and presence of HFSR) associated with prognosis (Table 3). In the univariate analysis, the presence of HFSR was the only prognostic factor associated with OS. Patients who developed HFSR demonstrated better OS compared to those who did not (5.74 vs. 3.12 months; HR: 0.41, 95% CI: 0.22–0.78; p = 0.005) (Fig. 3). A trend toward an OS advantage was observed in patients who received concomitant corticosteroids at the initiation of regorafenib, although this did not reach statistical significance (5.25 vs. 2.89 months; HR: 0.57, 95% CI: 0.31–1.05; p = 0.069). Univariate analysis of PFS did not identify any significant prognostic factors.

**Table 3:** Univariate analysis of variables associated with overall survival (OS) and progression-free survival (PFS).

Variable	Overall survival			Progression-free survival		
	Median OS, mo (95% CI)	HR (95% CI)	P	Median PFS, mo (95% CI)	HR (95% CI)	P
Age (years)						
< 65 (ref)	3.87 (1.95–5.80)	1.09 (0.56–2.08)	0.793	2.85 (2.02–3.68)	1.00 (0.53–1.91)	0.980
≥ 65	2.92 (1.36–4.48)			2.30 (1.63–2.96)		
Gender						
Female (ref)	5.25 (3.48–7.03)	1.30 (0.74–2.30)	0.352	3.12 (1.61–4.63)	1.24 (0.72–2.12)	0.430
Male	3.28 (1.69–4.87)			2.39 (1.99–2.80)		
ECOG-PS						
0–1 (ref)	4.83 (2.53–7.12)	1.27 (0.72–2.21)	0.397	2.85 (1.93–3.78)	1.11 (0.65–1.88)	0.700
2–3	3.81 (2.50–5.11)			2.30 (1.07–3.52)		
IDH status						
Mutated (ref)	5.78 (2.80–8.76)	1.53 (0.74–3.16)	0.240	3.25 (1.66–4.83)		
Wild	3.84 (2.77–4.91)			2.39 (1.57–3.22)	1.49 (0.75–2.97)	0.249
Initial surgery						
Complete resection (ref)	3.87 (1.71–6.03)	1.28 (0.74–2.22)	0.368	2.46 (1.59–3.33)	1.14 (0.67–1.94)	0.619
Partial resection or biopsy only	3.84 (2.83–4.85)			2.33 (1.32–3.34)		
Line of regorafenib treatment						
3 (ref)	3.81 (2.03–5.59)	0.60 (0.25–1.43)	0.245	2.43 (1.71–3.15)	0.71 (0.32–1.58)	0.406
4–5	3.84 (3.76–3.92)			3.22 (1.90–4.53)		
Initial dose of regorafenib						
80 mg (ref)	3.38 (2.35–4.41)	0.70 (0.40–1.24)	0.225	2.46 (1.49–3.42)	0.87 (0.50–1.51)	0.625
120—160 mg	5.45 (3.06–7.84)			2.30 (1.11–3.48)		
Dose reduction due to adverse events						
No (ref)	2.92 (1.77–4.07)	0.55 (0.30–1.02)	0.060	2.39 (2.04–2.74)		
Yes	5.45 (3.02–7.88)			3.12 (1.51–4.72)	0.66 (0.37–1.17)	0.157
Concomitant corticosteroid						
No (ref)	2.89 (1.73–4.04)	0.57 (0.31–1.05)	0.069	2.39 (1.96–2.82)	0.80 (0.45–1.39)	0.428
Yes	5.25 (3.46–7.04)			3.12 (2.09–4.14)		
HFSR						
Absent (ref)	3.12 (1.94–4.29)	0.41 (0.22–0.78)	0.005	2.26 (1.85–2.67)	0.63 (0.36–1.11)	0.111
Present	5.74 (3.78–7.41)			3.22 (2.35–4.08)		

ECOG-PS: Eastern Cooperative Oncology Group performance score, HFSR: Hand-foot skin reaction.

Since IDH-mutant gliomas are generally associated with a more favorable prognosis—and similar trends were observed in our cohort compared with IDH-wildtype glioblastoma—and given that the majority of our patients were IDH-wildtype glioblastoma (n = 55, 80.9%), we conducted Cox regression analysis restricted to the IDH-wildtype glioblastoma population to minimize potential bias in the interpretation of OS and PFS results (Supplementary Table S2). Consistent with the findings in the overall cohort, the presence of HFSR emerged as the only significant prognostic factor for OS (2.89 vs. 5.38 months; HR: 0.43, 95% CI: 0.21–0.86; p = 0.016). In addition, patients who developed HFSR demonstrated a trend toward improved PFS, although this did not reach statistical significance (2.13 vs. 3.45 months; HR: 0.54, 95% CI: 0.28–1.04; p = 0.065).

### Statistical power analysis

To assess whether the study sample size was sufficient to detect survival differences, we performed a post-hoc power analysis using the Schoenfeld method for Cox regression. The analysis was based on the total cohort (n = 68), the proportion of patients who developed HFSR (36.4%), and the number of observed survival events. For OS, the MDHR at 80% power was ~ 0.45 (protective direction, equivalent to ~ 2.23 in the adverse direction). The observed association (HR = 0.41) provided an achieved power of ~ 88%, indicating adequate sensitivity to detect the OS effect. For PFS, the MDHR at 80% power was ~ 0.46, whereas the observed effect (HR ≈ 0.63) corresponded to ~ 39% power, consistent with the non-significant result. Taken together, these findings suggest that the study was adequately powered to detect moderate-to-large effects on OS but underpowered to reliably capture smaller effects, particularly for PFS.

## Discussıon

This multicenter retrospective study evaluated real-world data on the efficacy and safety profile of regorafenib, as well as clinicopathological factors associated with survival, in patients with adult-type diffuse gliomas, including glioblastoma. Due to reimbursement regulations in our country, this study specifically investigated the use of regorafenib in second or subsequent recurrences. In our cohort of 68 patients, median PFS was 2.46 months and OS was 3.84 months. The ORR was 13%, and the DCR was 48%. Furthermore, the development of HFSR was the only clinicopathological factor associated with survival.

The REGOMA trial is a pivotal phase II study evaluating the efficacy and safety of regorafenib compared to lomustine in patients with glioblastoma following first recurrence^17^. In this study, all patients had glioblastoma histology and an ECOG-PS of 0–1, with 95% being IDH-wildtype. In the regorafenib cohort, median PFS was 2 months, median OS was 7.4 months, ORR was 5%, and DCR was 44%. The REGOMA-OSS study is a prospective observational study that included the largest number of patients to date^20^. This study included 190 patients with glioblastoma who received regorafenib after first recurrence. Similar to the REGOMA trial, all patients in this study had glioblastoma histology and an ECOG-PS of 0–1, with 92% being IDH-wildtype. The efficacy outcomes showed a median PFS of 2.6 months, a median OS of 7.9 months, an ORR of 13%, and a DCR of 39%. In studies evaluating regorafenib after first recurrence, including the REGOMA trial and others, the median PFS has been reported as approximately 2.0–2.7 months, while the median OS ranges from 7.0 to 10.0 months^17,20–22,24^. The better median OS reported in those studies compared to ours may be explained by the inclusion of patients who received regorafenib after first recurrence and had a better ECOG-PS. In contrast, in our cohort, all patients received regorafenib after second or subsequent recurrences, and only 42.6% had an ECOG-PS of 0–1. Since performance status tends to worsen with each recurrence in glioblastoma, particularly in later treatment lines, our study likely reflects the real-world setting more accurately.

The current literature includes only a limited number of studies investigating the use of regorafenib in second or subsequent recurrences of adult-type diffuse gliomas, including glioblastoma^25,26^. In a study by Tzaridis et al.^25^, which included 24 patients with high-grade gliomas (79.5% with glioblastoma, IDH-wildtype), the majority (87.5%) received regorafenib for second or subsequent recurrences and had poor performance status (62.5% had a Karnofsky Performance Status of 50–70%). The patient population was similar to ours, and the reported efficacy outcomes—median PFS of 2.1 months and median OS of 4.2 months—were consistent with our findings. In another study by Werner et al.^26^, which included high-grade gliomas in addition to glioblastoma, 73% of patients received regorafenib at the second or later recurrences. The study reported a median PFS of 2.6 months and a median OS of 6.2 months. Although the patient characteristics were very similar to our study, 97% of the patients had an ECOG-PS of 0–1, which may explain the longer median OS observed in this study compared to ours. Despite differences in the timing of regorafenib treatment and patient performance status, our results showed comparable ORR and DCR to those reported in the REGOMA trial^17^ and REGOMA-OSS study^20^ (ORR: 13% in our study, 5% in the REGOMA trial, 13% in the REGOMA-OSS study; DCR: 48% in our study, 44% in the REGOMA trial, 39% in the REGOMA-OSS study). These findings suggest that regorafenib maintains its therapeutic efficacy even in heavily pretreated patients with poorer performance status.

Safety analysis in our study showed that 80.9% of patients experienced any-grade regorafenib-related AEs, with grade 3–4 AEs occurring in 22.1% of patients. The incidence of grade 3–4 AEs was consistent with the REGOMA-OSS study ( 22.6%)^20^ but notably lower than the 56% rate reported in the REGOMA trial^17^. In our cohort, 5.9% of patients permanently discontinued regorafenib due to AEs, which is comparable to the 7% reported in the REGOMA trial^17^. Dose reductions were required in 32.4% of patients, which is higher than the 17% reported in the REGOMA trial but similar to the 36.8% observed in the REGOMA-OSS study^20^. Additionally, 23.5% of patients experienced treatment interruptions or delays, which is notably lower than the rates reported in the REGOMA trial (46%)^17^ and REGOMA-OSS study (44.7%)^20^. Importantly, no regorafenib-related deaths occurred in our cohort, consistent with the findings of both studies. These results suggest that regorafenib maintains a manageable safety profile, even in a real-world population characterized by more advanced disease, extensive prior treatment, and relatively poorer performance status. When evaluating HFSR in our cohort, any-grade events occurred in 36.4% of patients, with most being grade 1–2 and only one case (1.5%) of grade 3 toxicity. This incidence is comparable to that reported in the REGOMA trial^17^ (32% any grade; 10% grade 3–4) and the REGOMA-OSS study^20^ (1.6% grade 3–4), but notably lower than the grade 3–4 HFSR rates reported in the CORRECT^27^ (17%) and RESOURCE^28^ (13%) trials conducted in CRC and HCC populations, respectively.

In our univariate Cox regression analysis evaluating clinicopathological factors associated with OS and PFS, only the development of HFSR was found to be a significant predictor of improved OS (HR: 0.41; 95% CI: 0.22–0.78; p = 0.005). Consistent with our findings, Werner et al.^29^ reported in a cohort of 57 patients with recurrent glioblastoma, IDH-wildtype treated with regorafenib that HFSR developed in 25% of patients and was independently associated with improved OS (HR: 0.438; p = 0.039). Similarly, Tzaridis et al.^25^ showed that HFSR occurred in 30% of patients, with a significant survival advantage observed in those who developed HFSR (median OS: 6.7 vs. 2.6 months; p = 0.008). Several studies outside the glioblastoma setting have also provided evidence supporting this association. The CORRECT^27^ and RESOURCE trials^28^ demonstrated that HFSR—particularly when it occurs during the early phase of regorafenib treatment—serves as a prognostic marker in patients with CRC and HCC, respectively. In these studies, HFSR developed during the first cycle of regorafenib in 69% of patients in the CORRECT trial and 77% in the RESOURCE trial.

HFSR is a well-recognized adverse effect of multikinase inhibitors such as regorafenib and sorafenib, typically characterized by erythema, swelling, pain, and peeling of the skin—particularly on the palms and soles^30^. Mechanistically, the occurrence of HFSR has been attributed to regorafenib’s potent inhibition of VEGFR and PDGFR in skin capillaries, resulting in microvascular injury, inflammation, and subsequent skin toxicity^31^. This on-target but off-tumor toxicity is thought to reflect the extent of VEGFR inhibition and may serve as a surrogate marker for the anti-angiogenic activity achieved within the tumor microenvironment^32^. In glioblastoma, angiogenesis plays a pivotal role in tumor progression and is predominantly driven by VEGF overexpression, which leads to abnormal neovascularization and promotes an immunosuppressive microenvironment^33^. By blocking VEGFR and related pathways, regorafenib disrupts pathological vasculature and may enhance immune cell infiltration^34^. These effects are thought to underlie the clinical benefits observed in some patients. Thus, the occurrence of HFSR may indicate a subset of patients in whom VEGFR inhibition has reached a level sufficient to produce significant anti-tumor effects. Early development of HFSR may therefore reflect higher systemic drug exposure or increased pharmacodynamic sensitivity, ultimately translating into improved tumor control. An important clinical implication of these findings is that the development of HFSR should not prompt immediate treatment discontinuation or dose reduction.^35^. Instead, appropriate symptomatic management—such as the use of topical corticosteroids, keratolytics, and lifestyle modifications—is essential to enable patients to maintain regorafenib at the intended dose^30^. Notably, this management strategy is well established for HFSR induced by other multikinase inhibitors, such as sorafenib and sunitinib^35^, and can reasonably be extrapolated to regorafenib due to their similar mechanisms of action. In our study, as expected, patients who developed HFSR more frequently required dose reductions compared with those without HFSR (54.2% vs. 21.4%, p = 0.007). Moreover, although cumulative dosing could not be assessed, we observed that patients who initiated regorafenib at higher doses (120–160 mg) and those who required dose reductions due to adverse events tended to have longer survival. These findings suggest that dose reductions may not necessarily indicate treatment failure but could instead reflect adequate systemic exposure balanced with improved tolerability, thereby enabling patients to remain on therapy longer and potentially derive greater clinical benefit.

Our findings indicate that concomitant steroid use with regorafenib showed a trend toward improved OS, although the association was not statistically significant (HR for OS: 0.57; 95% CI: 0.31–1.05; p = 0.069). In the REGOMA trial^17^ and a retrospective study by Lombardi et al.^21^, concomitant steroid use with regorafenib was associated with poorer OS. However, these studies primarily included patients with first recurrence and good performance status (ECOG-PS 0–1), which differs from our study population. Additionally, the use of steroids during first-line treatment—particularly alongside concurrent radiotherapy and temozolomide—has been linked to worse survival outcomes^36^. In contrast, our data suggest that in more advanced disease and poorer performance status, steroid use may confer clinical benefit, potentially through the well-recognized effects of steroids in reducing peritumoral edema and alleviating neurological symptoms, thereby improving functional status and tolerability of oral treatment such as regorafenib^37^.

Our study has several limitations. First, the retrospective and multicenter design increases the risk of selection bias and may have introduced variability in histological and radiological evaluations across institutions. In addition, the inclusion of a histologically heterogeneous patient population—with approximately 80% diagnosed with glioblastoma, IDH-wildtype—limits comparability with studies employing the most recent WHO classification. However, around 90% of patients enrolled in the REGOMA trial^17^ and the REGOMA-OSS study^20^ were also IDH-wildtype, which is consistent with our cohort. Importantly, no phase II or III trials have yet evaluated regorafenib in glioblastoma strictly according to the 2021 WHO classification, underscoring the relevance of our findings. Although our study included a small number of IDH-mutant glioblastomas—no longer categorized as glioblastoma in the updated classification—the retrospective and multicenter design, particularly for patients diagnosed before 2021, limited the ability to uniformly reclassify cases. Nevertheless, the inclusion of these patients reflects the heterogeneity commonly encountered in real-world clinical practice and thereby enhances the generalizability of our findings. To further minimize potential bias, we additionally performed subgroup analyses restricted to IDH-wildtype glioblastomas. Consistent with the overall cohort, HFSR remained the only significant prognostic factor for OS, while a non-significant trend toward improved PFS was also observed.

Second, although the HFSR group included a somewhat higher proportion of ECOG 0–1 patients (54.2% vs. 38.1%, p = 0.206), which could theoretically contribute to better survival, multivariable Cox regression including ECOG and other covariates confirmed that HFSR was the only independent predictor of OS. This finding highlights the robustness of the association between HFSR and survival, independent of baseline ECOG performance status.

Another limitation is the lack of comprehensive MGMT promoter methylation data. Due to reimbursement restrictions, testing was performed in only a limited subset of patients (n = 17). Although MGMT promoter methylation is a well-established prognostic and predictive biomarker in glioblastoma, the primary aim of our study was to evaluate the efficacy and safety of regorafenib. Taken together, MGMT status was not included in the analyses, and this should be considered when interpreting the results.

Furthermore, the modest sample size may limit the generalizability of our findings. To address this, we conducted a post-hoc statistical power analysis, which indicated sufficient sensitivity to detect moderate-to-large effects on OS, whereas smaller effects—particularly for PFS—were less reliably captured. Thus, while the observed association between HFSR and OS appears statistically robust, confirmation in larger, prospective cohorts is warranted.

Finally, we did not assess the timing of HFSR onset during regorafenib treatment, as our initial focus was on identifying prognostic factors. Nevertheless, given that regorafenib was administered for a median of 2 cycles (range: 1–7) in our cohort, it is likely that HFSR developed during the early phase of treatment. This is consistent with findings from the CORRECT^27^ and RESOURCE^28^ trials, in which early-onset HFSR was identified as a prognostic marker. Similarly, in our study, patients who developed HFSR—likely in the early phase—showed better OS, supporting the potential role of early HFSR development as a predictive marker of treatment benefit.

## Conclusions

This multicenter study includes the largest real-world cohort to date evaluating regorafenib in patients with adult-type diffuse gliomas, including glioblastoma at second or later recurrences. Regorafenib was generally well tolerated, and the pattern of adverse events was similar to those reported in earlier studies. Although survival outcomes were modest, the absence of a standardized treatment option in this setting highlights regorafenib as a potentially valuable therapeutic choice. Importantly, our findings suggest that the early onset of HFSR is associated with improved OS, indicating its potential role as a clinical marker of treatment benefit. Recognizing HFSR early may help clinicians continue therapy confidently in appropriate patients. Given the exploratory nature of our results, prospective randomized trials are needed to validate these findings and guide future treatment strategies for this difficult-to-treat population.

## Supplementary Information

Supplementary Information.

## Data Availability

Due to ethical considerations, the datasets generated or analyzed during this study are not publicly available. Nonetheless, they can be obtained from the corresponding author (S.C.E) upon reasonable request.
