# Supplementary material for: Prognostic impact of hand-foot skin reaction in regorafenib-treated adult-type diffuse gliomas: A multicenter Turkish Oncology Group study
Source: Sci Rep. 2025 Oct 16;15:36175. doi: 10.1038/s41598-025-19899-3 (PMC12533162; doi:10.1038/s41598-025-19899-3)
Supplement: Supplementary file 1 — Supplementary Information. [file 41598_2025_19899_MOESM1_ESM.pdf]

Characteristics	HFSR Absent n=42 (63.6%)	HFSR Present n=24 (36.4%)	P*
**Age**			
<65 years	33 (78.6)	19 (79.2)	0.955
≥ 65 years	9 (21.4)	5 (20.8)	
**Gender**			
Female	19 (45.2)	8 (33.3)	0.344
Male	23 (54.8)	16 (66.7)	
**ECOG-PS at initiation of regorafenib**			
0-1	16 (38.1)	13 (54.2)	0.206
2-3	26 (61.9)	11 (45.8)	
**Histology**			
Glioblastoma, IDH-wild type	34 (81.0)	19 (79.2)	0.348
Glioblastoma, IDH-mutant#	3 (7.1)	4 (16.7)	
Astrocytoma, IDH-mutant, grade 3	3 (7.1)	1 (4.2)	
Oligodendroglioma, IDH-mutant, grade 3	2 (4.8)	0 (0.0)	
**IDH status**			
Mutated	8 (19.0)	5 (20.8)	0.861
Wild	34 (81.0)	19 (79.2)	
**Type of initial surgery**			
Complete resection	22 (52.4)	15 (62.5)	0.426
Partial resection or biopsy only	20 (47.6)	9 (37.5)	
**First-line treatment**			
CRT (with TMZ) followed by adjuvant TMZ	40 (95.2)	22 (91.7)	0.716
CRT only	1 (2.4)	2 (8.3)	
RT followed by adjuvant TMZ	1 (2.4)	0 (0.0)	
**Line of regorafenib therapy**			
3	37 (88.1)	19 (79.2)	0.330
4-5	5 (11.9)	5 (20.8)	
**Surgery before regorafenib**			
No	42 (100.0)	23 (95.8)	0.364
Yes	0 (0.0)	1 (4.2)	
**Radiation before regorafenib**			
No	31 (73.8)	22 (91.7)	0.111
Yes	11 (26.2)	2 (8.3)	
**Concomitant corticosteroid**			
No	17 (40.5)	8 (33.3)	0.565
Yes	25 (59.5)	16 (66.7)	
**Initial dose of regorafenib**			
80 mg	23 (54.8)	15 (62.5)	0.932
120 mg	13 (31.0)	6 (25.0)	
160 mg	6 (14.3)	3 (12.5)	
**No. of regorafenib cycle, median (min-max)**	2 (1-7)	3 (1-6)	0.054
**Dose reduction due to adverse events**	9 (21.4)		
No	33 (78.6)	11 (45.8)	0.007
Yes	9 (21.4)	13 (54.2)	
**No. of treatment lines after regorafenib**			
Regorafenib is ongoing	7 (16.7)	3 (12.5)	0.065
Regorafenib is the last line	33 (78.6)	16 (66.7)	
Received 1 line of treatment after regorafenib	1 (2.4)	5 (20.8)	
Received 2 line of treatment after regorafenib	1 (2.4)	0 (0.0)	

CRT: Chemoradiotherapy, ECOG-PS: Eastern Cooperative Oncology Group performance score, HFSR: Hand-foot skin reaction, RT: Radiotherapy, TMZ: Temozolomid

*Chi-square test or Mann-Whitney U test

Included as glioblastoma, IDH-mutant according to previous WHO classifications.

Supplementary Table S1. Baseline clinicopathological characteristics of patients according to the presence of hand–foot skin reaction (HFSR)

	Overall Survival			Progression-free Survival		
**Variable**	**Median OS, mo (95% CI)**	**HR (95% CI)**	**P**	**Median PFS, mo (95% CI)**	**HR (95% CI)**	**P**
**Age (years)**						
<65 (ref)	3.38 (2.21-4.55)			2.46 (1.71-3.21)		
≥65	4.83 (0.10-9.56)	0.78 (0.34-1.78)	0.564	2.07 (1.63-2.50)	0.77 (0.34-1.74)	0.535
**Gender**						
Female (ref)	4.83 (2.50-7.15)			2.99 (1.47-4.50)		
Male	3.28 (1.81-4.75)	0.91 (0.48-1.71)	0.772	2.39 (1.59-3.20)	0.87 (0.48-1.60)	0.675
**ECOG-PS**						
0-1 (ref)	4.83 (2.29-7.36)			2.85 (2.14-3.56)		
2-3	2.89 (1.48-4.29)	1.36 (0.74-2.51)	0.308	2.07 (1.42-2.71)	0.98 (0.55-1.77)	0.972
**Initial surgery**						
Complete resection (ref)	3.38 (1.68-5.07)			2.39 (1.51-3.28)		
Partial resection or biopsy only	3.84 (2.65-5.03)	1.29 (0.69-2.42)	0.413	2.95 (1.83-4.07)	1.04 (0.57-1.89)	0.880
**Line of regorafenib treatment**						
3 (ref)	3.28 (2.01-4.55)			2.33 (1.86-2.79)		
4-5	5.38 (2.11-8.65)	0.38 (0.13-1.10)	0.065	3.22 (1.16-5.28)	0.49 (0.19-1.25)	0.127
**Initial dose of regorafenib**						
80 mg (ref)	3.12 (1.75-4.49)			2.39 (1.36-3.43)		
120 - 160 mg	5.38 (1.83-8.93)	0.93 (0.49-1.75)	0.823	2.13 (0.98-3.28)	1.14 (0.61-2.15)	0.670
**Dose reduction due to adverse events**						
No (ref)	2.89 (1.53-4.24)			2.26 (1.78-2.74)		
Yes	5.45 (2.58-8.32)	0.56 (0.29-1.11)	0.094	3.12 (1.61-4.62)	0.63 (0.33-1.20)	0.159
**Concomitant corticosteroid**						
No (ref)	2.89 (1.16-4.62)			2.39 (1.82-2.96)		
Yes	3.94 (1.23-6.65)	0.60 (0.30-1.18)	0.135	2.85 (1.75-3.96)	0.76 (0.40-1.42)	0.387
**HFSR**						
Absent (ref)	2.89 (1.76-4.01)			2.13 (1.84-2.42)		
Present	5.38 (3.19-7.58)	0.43 (0.21-0.86)	0.016	3.45 (2.56-4.33)	0.54 (0.28-1.04)	0.065

ECOG-PS: Eastern Cooperative Oncology Group performance score, HFSR: Hand-foot skin reaction

Supplementary Table S2. Univariate analysis of variables associated with overall survival (OS) and progression-free survival (PFS) in patients with IDH-wildtype glioblastoma
